# Functional acclimation across microgeographic scales in *Dodonaea viscosa*

**DOI:** 10.1093/aobpla/ply029

**Published:** 2018-05-11

**Authors:** Zdravko Baruch, Alice R Jones, Kathryn E Hill, Francesca A McInerney, Colette Blyth, Stefan Caddy-Retalic, Matthew J Christmas, Nicholas J C Gellie, Andrew J Lowe, Irene Martin-Fores, Kristine E Nielson, Martin F Breed

**Affiliations:** 1 School of Biological Sciences and the Environment Institute, University of Adelaide, North Terrace, Adelaide, SA, Australia; 2 Sprigg Geobiology Centre and School of Physical Sciences, University of Adelaide, Adelaide, SA, Australia; 3 Department of Medical Biochemistry and Microbiology, Uppsala University, Uppsala, Sweden; 4 National Museum of Natural Sciences, Spanish National Research Council, Madrid, Spain

**Keywords:** Adaptive capacity, common garden experiment, functional traits, microgeography, plasticity, shrubs, South Australia

## Abstract

Intraspecific plant functional trait variation provides mechanistic insight into persistence and can infer population adaptive capacity. However, most studies explore intraspecific trait variation in systems where geographic and environmental distances co-vary. Such a design reduces the certainty of trait–environment associations, and it is imperative for studies that make trait–environment associations be conducted in systems where environmental distance varies independently of geographic distance. Here we explored trait variation in such a system, and aimed to: (i) quantify trait variation of parent and offspring generations, and associate this variation to parental environments; (ii) determine the traits which best explain population differences; (iii) compare parent and offspring trait–trait relationships. We characterized 15 plant functional traits in eight populations of a shrub with a maximum separation ca. 100 km. Populations differed markedly in aridity and elevation, and environmental distance varied independently of geographic distance. We measured traits in parent populations collected in the field, as well as their offspring reared in greenhouse conditions. Parent traits regularly associated with their environment. These associations were largely lost in the offspring generation, indicating considerable phenotypic plasticity. An ordination of parent traits showed clear structure with strong influence of leaf area, specific leaf area, stomatal traits, isotope δ^13^C and δ^15^N ratios, and N_area_, whereas the offspring ordination was less structured. Parent trait–trait correlations were in line with expectations from the leaf economic spectrum. We show considerable trait plasticity in the woody shrub over microgeographic scales (<100 km), indicating it has the adaptive potential within a generation to functionally acclimate to a range of abiotic conditions. Since our study shrub is commonly used for restoration in southern Australia and local populations do not show strong genetic differentiation in functional traits, the potential risks of transferring seed across the broad environmental conditions are not likely to be a significant issue.

## Teaser

We studied a native Australian shrub—*Dodonaea viscosa*, or sticky hop bush—in the wild and in a gardening experiment and found that the species can readily adapt to different environments. Our findings are interesting because the plants we used came from sites with quite different environmental conditions, although they were only short distances apart. Our findings indicate that the potential risks associated with moving plants between sites with different environmental conditions are not likely to cause negative outcomes for restoration projects using this species, which is commonly used for restoration in southern Australia.

## Introduction

Exploring variation in functional traits across environmental gradients provides mechanistic insights into the persistence and function of widespread species (Ramírez-Valiente *et al.* 2010; Bolnick *et al.* 2011; Funk *et al.* 2017). Similarly, such studies can be combined with fitness measurements to infer adaptive capacity of populations, and can therefore be used to predict the success of plant populations and derive seed sourcing options for revegetation under climate change (Laughlin 2014; Breed *et al.* in press). However, most studies that explore intraspecific trait variation do so in systems where geographic and environmental distances co-vary (e.g. climate distance correlates with geographic distance) (Hulshof *et al.* 2013; Carlson *et al.* 2016). Such co-variation makes it difficult to determine the specific abiotic driver(s) of trait variation and limits the utility of this type of research (Meirmans 2015; Caddy-Retalic *et al.* 2017). While there are statistical ways to account for this issue *post hoc* (Dormann *et al.* 2007; Hothorn *et al.* 2011), it remains preferable to associate environmental and trait variation in systems where environmental distance varies independently of geographic distance.

Studies that seek to make conclusions on plant adaptive capacity can usefully explore trait variation across environmental gradients at microgeographic scales (e.g. tens of kilometres for trees and shrubs). These smaller spatial scales provide greater potential for gene flow to occur between populations, enabling a test of whether the influx of genes from non-local populations is ‘swamping’ local gene pools. An influx of non-local genes could increase trait variation, and potentially prevent local adaptation (Richardson *et al.* 2014). Including a common garden component to these studies helps to control for the environmental influence on traits, allowing an understanding of whether trait variation is under genetic vs. plastic control (Poorter *et al.* 2016). When studies are conducted in such a way, it should be possible to quantify species trait variability in the presence of gene flow. Similarly, such studies (supported by reproductive traits), and combined with fitness measurements, can be used to infer adaptive capacity of populations. This information is useful in a conservation context as it can help to predict the impacts of habitat fragmentation, which tends to reduce interpopulation gene flow, disrupt plant mating systems and reduce population sizes (Young *et al.* 1996; Lowe *et al.* 2005, 2015; Vranckx *et al.* 2012; Breed *et al.* 2013, 2015). These impacts can each reduce the adaptive capacity to climate change of plant populations (Breed *et al.* 2012; Christmas *et al.* 2016b). However, there are surprisingly few fine-scale studies of trait variation reported (Richardson *et al.* 2014).

**Figure 1.**
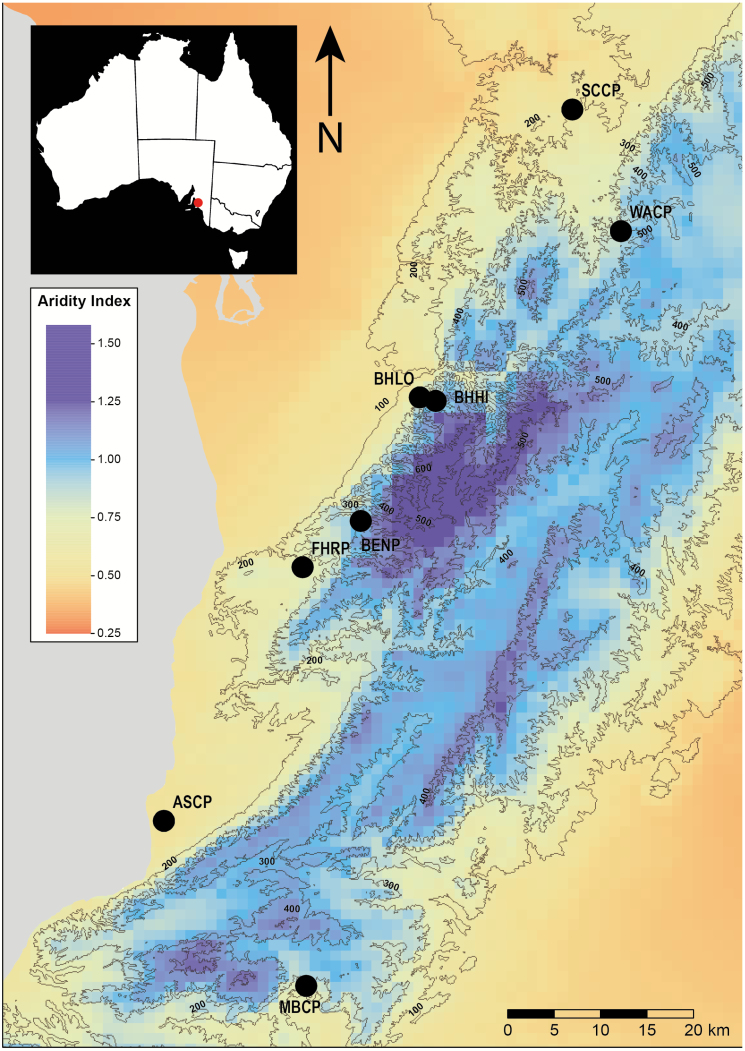
Locations of populations on the Fleurieu Peninsula, South Australia (black filled circles). The context layer is the aridity index (AI), where lower values represent the more arid sites. Contour lines at 100 m each.

*Dodonaea viscosa* (Sapindaceae) (hereafter Dodonaea) occurs in Australia across a broad and variable environment, where it displays clinal variation in a number of functional traits (Guerin *et al.* 2012; Guerin and Lowe 2013; Hill *et al.* 2015; Baruch *et al.* 2017). In the Mt Lofty Ranges (South Australia), Dodonaea occurs continuously across a short latitude span (34.6 to 35.5°S = ca. 100 km), but across a variety of elevations (from sea level up to 300 m). This short geographic distance displays large variation in aridity, precipitation and temperature (Fig. 1; Table 1). Dodonaea also displays low levels of population genetic structure across this region, indicating considerable gene flow (as observed in other plant species; McCallum *et al.* 2014), but selection appears to have been sufficiently strong to maintain adaptive genetic variation (Christmas *et al.* 2016a, 2017). To the best of our knowledge, no detailed trait-based studies have been conducted in any plant taxon in this region. As such, this environment is an excellent system to study functional trait variation, and investigate whether trait variation still occurs despite likely gene flow across population in this area.

**Table 1.** List of environmental variables and parent and offspring traits that were measured and analysed in this study. The acronyms and units employed throughout the text, figures and tables are shown.

Variable	Type	Acronym	Measurement units	Parents		Offspring	
				Tested	Sample size	Tested	Sample size
Elevation	Environmental		m	NA	NA	NA	NA
Aridity index	Environmental	AI	NA	NA	NA	NA	NA
Mean annual precipitation	Environmental	MAP	mm	NA	NA	NA	NA
Mean annual temperature	Environmental	MAT	°C	NA	NA	NA	NA
Soil nitrogen content	Environmental	N	kg ha^−1^	NA	NA	NA	NA
Soil phosphorus content	Environmental	P	kg ha^−1^	NA	NA	NA	NA
Clay content	Environmental		%	NA	NA	NA	NA
Leaf area	Trait	LA	cm^2^	√	400	√	80
Specific leaf area	Trait	SLA	cm^2^ g^−1^	√	400	√	80
Stomatal size	Trait	SS	µm	√	80	√	80
Stomatal density	Trait	SD	# per mm^2^	√	80	√	80
Carbon isotope ratio	Trait	δ^13^C	NA	√	80	√	80
Nitrogen isotope ratio	Trait	δ^15^N	NA	√	80	√	80
Leaf nitrogen content (on mass basis)	Trait	N_mass_	%	√	80	√	80
Leaf nitrogen content (on area basis)	Trait	N_area_	gN m^−2^	√	80	√	80
Leaf carbon/nitrogen ratio	Trait	C:N	NA	√	80	√	80
Wood density	Trait	WD	g cm^−3^	√	80	NA	NA
Seed mass	Trait	SW	mg per 50 seeds	√	80	NA	NA
Germination	Trait	GERM	%	NA	NA	√	400
Plant height	Trait	HEIGHT	cm	NA	NA	√	1600
Relative growth rate	Trait	RGR	day^−1^	NA	NA	√	1600
Leaf thickness	Trait	THICK	µm	NA	NA	√	80

Here we assessed trait variability of Dodonaea adult field populations, and compared these data with trait values of offspring grown in a greenhouse common garden trial. We measured functional traits associated with plant architecture, competitive ability and dispersal capacity (i.e. height, wood density, growth rate and seed mass). We also characterized leaf traits that modulate leaf temperature, transpiration and photosynthesis, as well as resource use efficiency on short and long timescales (i.e. specific leaf area (SLA), nitrogen content per unit mass and area, carbon and nitrogen isotope ratios, stomatal size and density). We used these data to explore the following study goals: (i) quantify trait variability of parents and offspring, and link this variation to the parental environments; (ii) determine which traits best explain population differences; and (iii) compare parent and offspring trait–trait relationships.

## Methods

### Study species and field sampling

Dodonaea is a 1–4 m tall woody shrub, with upright, narrow, tough and sticky leaves covered by reflective wax. Pollination is by wind and insects, and seeds are dispersed by wind and ants. Dodonaea is widely distributed throughout the southern half of Australia, predominantly on well-drained soils, and it can re-sprout after fire (Hodgkinson 1998). Locally, it forms sparse to dense cover in shrublands and in open woodlands as a recognizable shrub layer (Hodgkinson and Oxley 1990).

We selected eight parent populations in the Mt Lofty Ranges, South Australia. Local climates varied considerably due to differences in altitude and its effects on precipitation, aridity and air temperature. All populations (henceforth designated by their initials in Fig. 1 and Table 1) were located in remnant vegetation within nature reserves (Fig. 1; Table 1). Between October and December 2015, we collected five sun-exposed terminal branch segments from 10 non-neighbouring parent plants (henceforth families), separated by >5 m, which were stored at 4 °C until processing. Concurrently, we also harvested mature fruits from across the canopy of each parent plant, which were stored in paper bags in dry, room temperature conditions.

### Parent populations handling and traits

We measured 11 traits in the parent cohort for our analyses (Table 1). Five of the youngest, fully expanded leaves of each parent plant were excised to estimate leaf area by scanning and measuring with ImageJ (Schneider *et al.* 2012). Each leaf was then oven dried at 65 °C for 48 h and weighed. Specific leaf area (the ratio of leaf area to mass) was calculated (*n* = 400; 5 leaves × 10 parent plants (families) × 8 populations). Wood density was determined by the dimensional method, described in Pérez-Harguindeguy *et al.* (2013), from the proximal end of one branch segment per parent from each population. Uniform ca. 3 cm segments (close to cylindrical and care was taken to avoid rough sections) were cut and their length and diameter measured with digital callipers, to obtain volume. These segments were then oven dried at 85 °C for 72 h and weighed. Wood density is the ratio of dry weight and volume (*n* = 80; 10 families × 8 populations). Stomatal size and density were determined on 1 cm^2^ leaf segments excised from the middle of the abaxial lamina of one leaf per parent (*n* = 80). The cuticle preparations and measurements were the same as described in Hill *et al.* (2015).

Carbon and nitrogen isotope (δ^13^C; δ^15^N) ratios and element content (C_mass_; N_mass_) were determined on dried leaves of parent plants (*n* = 80). Leaves were ground in 2 mL screw cap micro centrifuge tubes with a Qiagen TissueLyzer (Dusseldorf, Germany) adaptor for a Retsch ball mill (Mixer Mill 400, Haan, Germany). Ground material (ca. 2.5 mg) was weighed in tin capsules. Samples were analysed at the University of Adelaide on a EuroVector Euro EA inline (Pavia, Italy) with a Nu Instruments Continuous Flow Isotope Ratio Mass Spectrometer (CF-IRMS, Wrexham, UK). Internal isotope standards run alongside were glycine and glutamic acid. Certified reference material for elemental concentration was triphenyl amine (TPA; C:N). The uncertainty of ^13^C and ^15^N measurements was less than ±0.09 and ±0.12 ‰, respectively. The C:N ratio was assessed as well as the N content per unit leaf area (N_area_), which was calculated from N_mass_ and SLA. Fruits were manually crushed to liberate seeds, and seeds were weighed in sets of 50 per parent to obtain mean seed weight.

### Offspring population handling and traits

We measured 13 offspring traits, 9 of which were also measured in the parent plants (Table 1). Seed dormancy was broken by soaking seeds for 5 min in recently boiled water (Baskin *et al.* 2004). Five replicates of 20 seeds per family were set to germinate on moist filter paper in Petri dishes. These dishes were arrayed on a greenhouse bench, moistened as required with demineralized water, and their position in the greenhouse randomized fortnightly. The protrusion of the radicle indicated germination, and its presence was scored three times per week over 2 months (from February to April 2016), and these seedlings were then discarded. During this germination trial, mean air temperature was 16.5 °C (range: 3.7–40.5 °C) and mean relative humidity was 77.3 % (range: 21.1–99.9 %) recorded with a Senonics Minnow data logger (MicroDAQ, USA). Shade cloth covered the roof of the greenhouse to decrease photosynthetic active radiation (PhAR) irradiance, which was between one-third and half of that outside as measured with a quantum sensor Q 34327 and LI-1400 data logger (Li-COR, Lincoln, NE, USA).

In January 2016, 20 replicates per family per population (20 × 10 × 8; *n* = 1600) were sown each with five seeds over commercial native potting mix (Grow Better) in standard forestry tubes (5 × 5 × 12 cm; see **Supporting Information—Fig. S1** for plant images). These were distributed randomly into trays whose position in the greenhouse was randomized fortnightly. Spray watering was provided three times per week, for 10 min at 3-h intervals. Pots were fertilized once with 0.2 g slow release Osmocote Native Mix. To minimize selection for fitness, if more than one seed germinated per pot, the most central seedling was chosen and the rest were removed. Seedling height was scored twice: (*t*_1_) at 96 days after sowing and (*t*_2_) on 173 days after sowing. Instantaneous relative growth rate was calculated as relative growth rate (RGR) = (log_e_HEIGHT_2_ − log_e_HEIGHT_1_)/(*t*_2_ − *t*_1_). We employed the non-destructive, seedling height as a proxy for plant weight. Survival on both dates was recorded.

In September 2016 (234 days after sowing), we sorted the seedlings from every family by height and selected the four closest to the median (*n* = 320 seedlings: 4 replicates × 10 families × 8 populations). These were transplanted to larger polyethylene pots (8.5 × 8.5 × 17.5 cm) with the same potting mix, and transferred to a greenhouse bench where mean air temperature was 17.89 °C (range: 4.75–31.53 °C) and mean relative humidity was 70.80 % (range: 35.23–90.25 %). Photosynthetic active radiation was about half of that outside, and both temperature and PhAR were monitored and recorded as above. Plants were watered for 10 min twice per day, four times per week, and top-dressed with 0.6 g of the same fertilizer. Thirteen months after sowing, 80 young but fully expanded leaves (one leaf per randomly selected individual per family) were excised to measure SLA, stomatal density and size, as well as isotope ratios and elemental content as above. Leaf thickness of offspring was determined on 80 mature leaves (10 families × 8 populations) as the mean from 10 measurements per leaf at ~1 mm from the mid-vein at the centre of the leaf on the same 80 individuals.

### Environmental variables

We included seven environmental variables in our analyses, including the aridity index (AI), mean annual precipitation (MAP), mean annual temperature (MAT), and soil nitrogen (N), phosphorus (P) and clay content (see Table 2 for further details). Environmental data were sourced from the Atlas of Living Australia at 0.01° (~1 km) resolution (http://www.ala.org.au; accessed 15 December 2016) (Williams *et al.* 2012). Geographic linear distances between populations were estimated from GPS coordinates and suitable software (http://www.boulter.com/gps/distance/).

**Table 2.** Geographic coordinates, elevation, climate and soil variables of the eight *Dodonaea viscosa* subsp. *angustissima* study populations. C.P. = Conservation Park; N.P. = National Park; R.P. = Recreation Park; AI = aridity index = an inverse scale of aridity, where high values indicate less arid climates; MAP = mean annual precipitation; MAT = mean annual temperature; N and P = pre-European plant available soil N and P stores.

Population	Code	Latitude	Longitude	Elevation (m)	AI	MAP (mm)	MAT (°C)	N (kg ha^−1^ × 10^3^)	P (kg ha^−1^)	Clay (%)
Aldinga Scrub C.P.	ASCP	−35.29	138.46	17	0.55	536	16.2	12.83	21.45	9.84
Belair N.P.	BENP	−35.00	138.65	267	0.95	524	15.3	8.81	12.63	34.53
High elevation Black Hill C.P.	BHHI	−34.89	138.72	244	1.08	603	14.7	8.14	11.73	28.88
Low elevation Black Hill C.P.	BHLO	−34.89	138.71	163	0.68	603	15.8	8.14	11.73	28.88
Flagstaff Hill R.P.	FHRP	−35.05	138.59	153	0.67	493	16.1	8.64	9.02	34.91
Mt. Billy C.P.	MBCP	−35.45	138.60	260	0.94	852	14.5	9.57	14.45	32.28
Sandy Creek C.P.	SCCP	−34.61	138.86	196	0.60	533	15.8	7.51	13.15	28.29
Warren C.P.	WACP	−34.73	138.90	299	0.88	646	14.7	8.27	11.12	25.42
ANOVA *F*_(1, 6)_				10.8	40.58	2.44	8.5	0.21	0.64	0.43
ANOVA *P*				<0.05	<0.05	ns	<0.05	ns	ns	ns

### Data analysis

We used one-way ANOVAs to test for environmental differences and parent and offspring trait differences between populations. We used linear mixed-effects models and ordinations to explore variation in traits, trait–trait correlations and trait–environment associations. All traits were treated as continuous response variables. **Supporting Information—Appendix S1** describes our statistical analyses in more detail.

To reduce redundancy in the environmental predictor variables, we ran a principal component analysis (PCA) to obtain an integrated, composite environmental ordination for each population. Since soil N, P and clay content were very different at one population (ASCP; Table 1), the PCA was only run on climatic variables, which were all strongly associated with elevation. Parent mean trait and offspring traits were correlated against their position along the environmental PCA1 and PCA2 axes. We also ran PCAs of parent and offspring functional traits to assess their relative influence within these two cohorts. To test for the association and significance of the multidimensional parent and offspring trait spaces, their respective Euclidean interpopulation distance matrices were computed and then correlated with the Mantel test (Mantel 1967). To account for the effect of environmental differences and geographical distances, partial Mantel tests (Smouse *et al.* 1986) were employed. Multivariate analyses were done in PC-ORD V6.0 (McCune and Mefford 2011).

To explore trait–trait correlations, we ran linear regressions in SYSTAT (2002) for each pair of traits within parent and offspring cohorts. Traits shared between parents and offspring were also regressed. We used sequential Bonferroni adjustment of the P threshold for multiple testing. We fitted linear mixed-effects models in R (R Core Team 2017) using the package lme4 (Bates *et al.* 2015) to test for differences in offspring relative growth rate (Gaussian distribution) and height (Gamma distribution) between populations, including family as a random effect nested within population. We generated *P* values and 95 % confidence intervals for the effect of population on relative growth rate and height using parametric bootstrapping methods (1000 iterations) implemented in the R package ‘afex’ (Singmann *et al.* 2017).

## Results

### Environmental and geographic distances

Dodonaea populations were separated by 1.4 to 96.8 km (Fig. 1; see **Supporting Information—Table S1**) at elevations that ranged from 17 to 299 m above sea level (Table 1). As expected, elevation regulated local climates, where more arid and warmer sites were at lower elevations (ASCP, FHRP, BHLO, SCCP) and the less arid and cooler sites were at higher elevation (MBCP, BENP, BHHI, WACP). Aridity and air temperature were higher at sites below 200 m above sea level, while rainfall was greater at higher elevation sites (Table 1). Soil texture, N and P content did not differ between these two groups, but soils from ASCP were unlike the other populations, with considerable less clay and more nutrients (Table 1).

**Figure 2.**
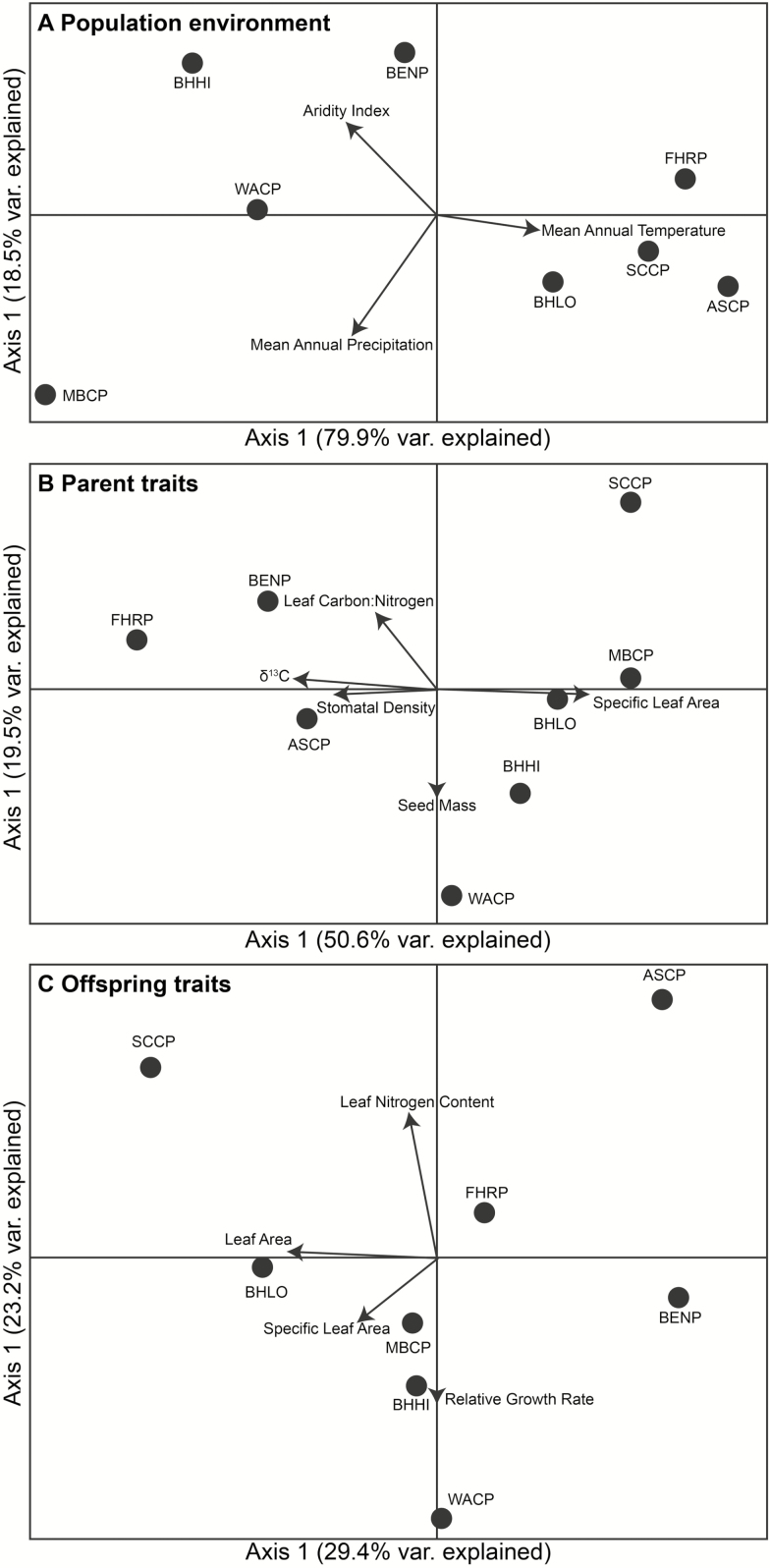
PCA ordination of population environment (A); variance explained PCA1 = 79.9 %; PCA2 = 18.5 %. PCA ordination of parent populations in trait space (B); variance explained PCA1 = 50.6 %; PCA2 = 19.5 %. PCA ordination of offspring populations in trait space (C); variance explained PCA1 = 29.4 %; PCA2 = 23.2 %. Arrows indicate the importance and trends of variables in the PCA. Only variables whose correlation with axes was >0.7 are shown.

The ordination of populations within environmental space clearly separated low and high elevation populations (Fig. 2A), where PCA1 was strongly correlated with all climatic variables **[see **Supporting Information—Table S2** ]**, and explained 79.9 % of the total variance. The AI and MAP were associated negatively with environmental PCA1 and MAT was associated positively with environmental PCA1. Environmental and geographical distances were uncorrelated in the ordination plot (Fig. 2A), confirmed by the lack of significant multivariate association between them (Table 2).

### Trait differences in parent and offspring cohorts

**Figure 3.**
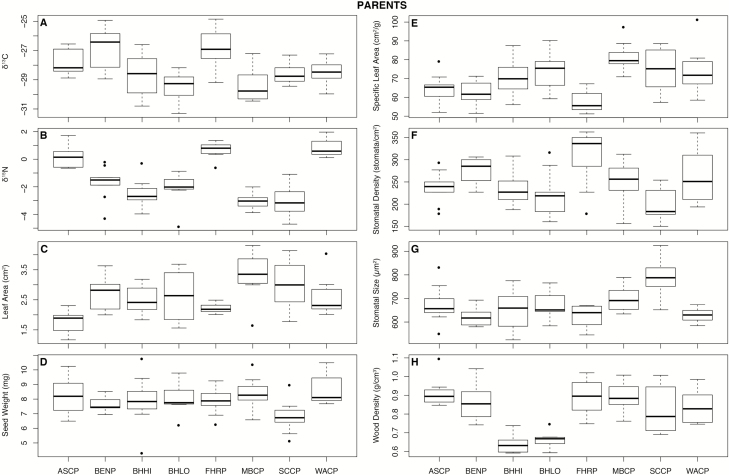
(A–H) Box plots of parent traits that display significant variation across populations.

**Figure 4.**
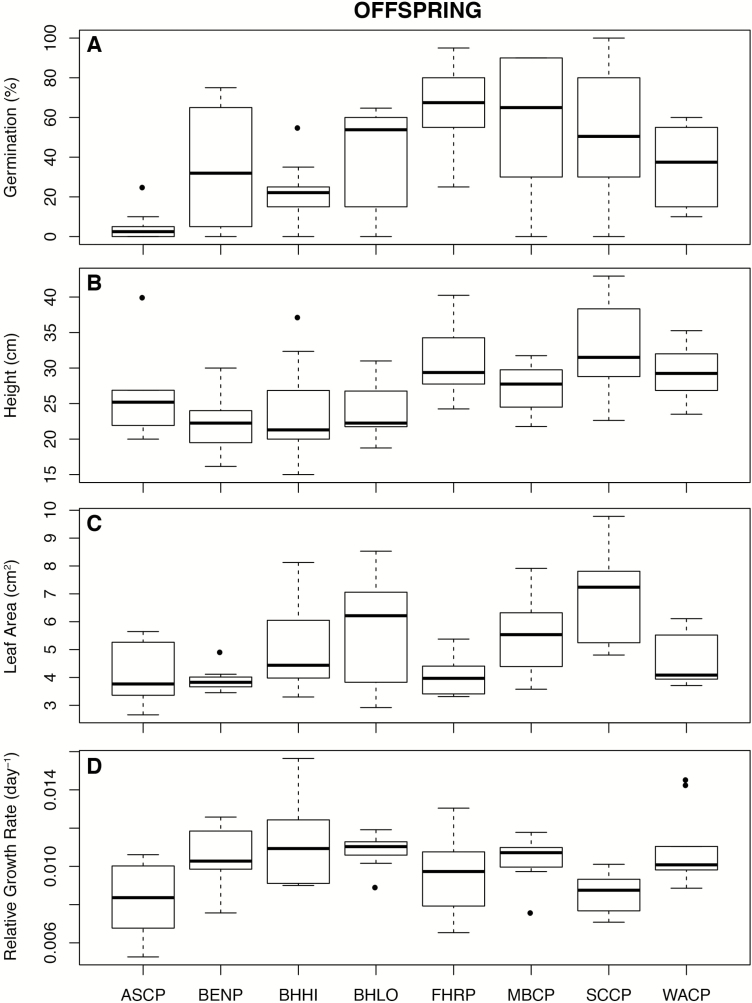
(A–D) Box plots of offspring traits that display significant variation across populations.

Apart from N_mass_, all traits differed significantly across parent populations (Fig. 3A–H; see **Supporting Information—Table S3**). Offspring differed only in leaf area, germination (Fig. 4A–D; **Supporting Information—Table S3**), height (χ^2^ = 35.02, *P* < 0.001; **Supporting Information—Fig. S2**) and relative growth rate (χ^2^ = 28.09, *P* < 0.001; see **Supporting Information—Fig. S2**). For height and relative growth rate, linear mixed-effects models (assuming normal error distribution) showed that family (random effect) explained very little variation in either trait. Survival on day 173 after sowing was notably low in ASCP and BHHI populations (50.5 and 62.5 %, respectively), where survival in all other populations was >90 %.

Mean leaf area and SLA were substantially greater in offspring than in parent populations (ca. 2.0- and 3.5-fold, respectively). Conversely, mean N_mass_, N_area_ and the δ^13^C ratio were higher in the parents (1.9-, 1.6- and 2.1-fold, respectively) (Table 2; Figs 3 and 4). Dodonaea germination was uneven, FHRP, MBCP and SCCP were the first to germinate after 26 days, and also displayed the highest germination percentages, whereas ASCP had the poorest germination (Fig. 4A).

### Parent and offspring trait–environment correlations

**Figure 5.**
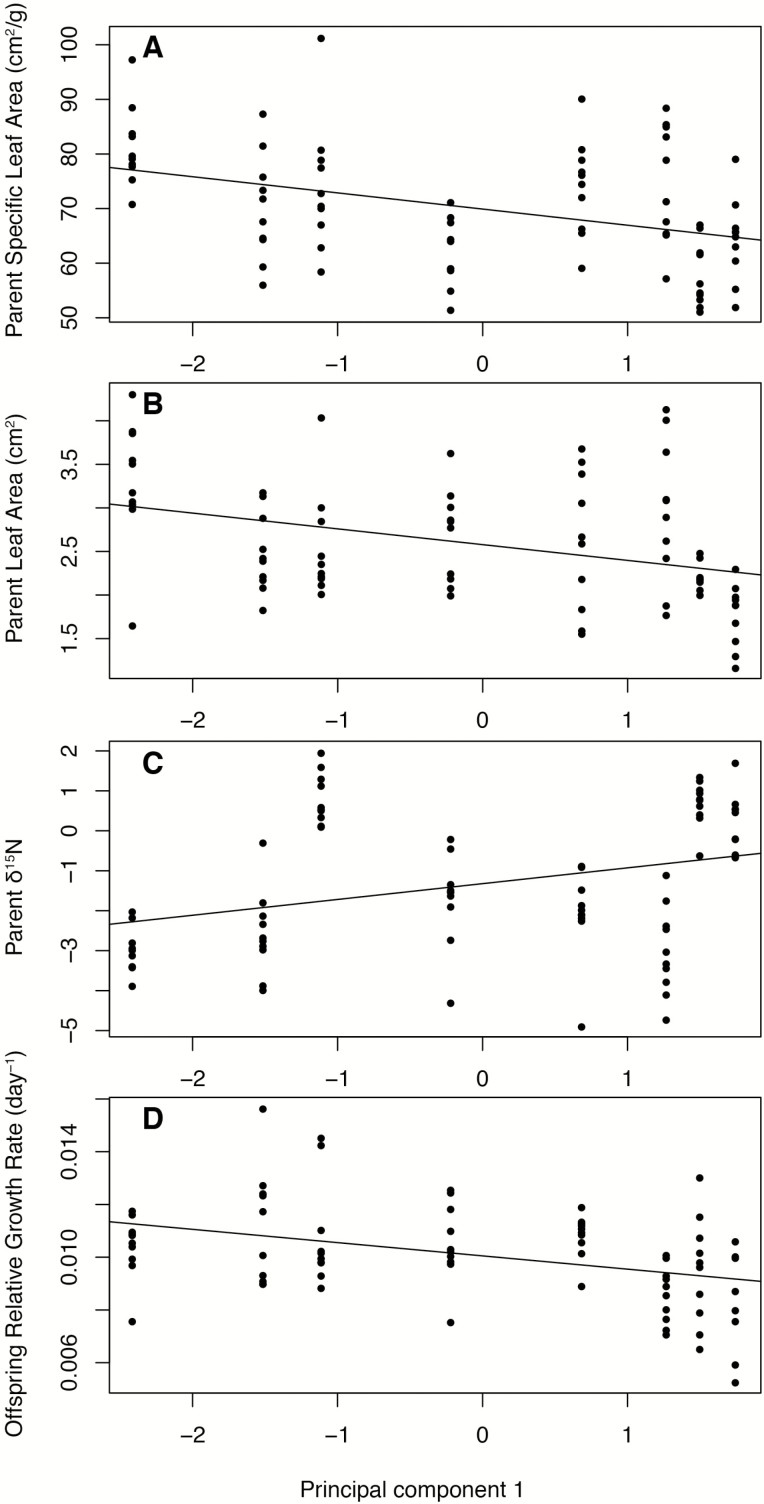
Scatterplots showing fitted linear models between traits and parent and offspring positions along the composite (AI, MAP, MAT) environmental axis PCA1. Only traits with significant regression coefficients after Bonferroni sequential adjustments are shown.

Traits of parent and offspring displayed distinctive correlations with environment PCA1 and PCA2 (Fig. 5; see **Supporting Information—Tables S4 and S5**). For parents, leaf area and SLA were negatively correlated to PCA1, implying that they increase with elevation, precipitation and lower aridity. The δ^15^N ratio was positively correlated to PCA1, indicating that it increased at lower elevation in warmer and more arid sites. In offspring, only relative growth rate was correlated to PCA1 (Fig. 5D; see **Supporting Information—Tables S4 and S5**).

### Parent and offspring in multi-trait space

The ordination of parent populations in trait space was quite different to the environmental space ordination that showed environmental disparity between high and low elevation populations (Fig. 2B). Leaf area, SLA, stomata size, stomata density, both isotope ratios and N_mass_ had the largest influence on the ordination along PCA1 (50.6 % of total variance explained), and only mean seed weight was associated to PCA2 (19.5 % of total variance explained) (Fig 2B; see **Supporting Information—Table S2**). A negative correlation between stomata size and stomata density was evident **[see **Supporting Information—Table S2** ]**. The ordination of offspring was less structured than parents as both ordination PCA axes captured equally low percentages of total variance (29.4 and 23.5 %, respectively). Leaf area, SLA, leaf thickness and stomata density defined the ordination along PCA1, whereas relative growth rate and N_mass_ defined the ordination along PCA2 (Fig. 2C; see **Supporting Information—Table S2**). In this offspring trait ordination, populations from cooler and wetter sites appeared to be more clustered than those from warmer and drier sites. The distinct ordinations of parent and offspring populations in trait space were confirmed by the lack of correlation between their respective distance matrices, even when controlling for geographic and environmental distances using a partial Mantel test (Table 3).

**Table 3.** Multivariate correlations between environmental and geographic distances, parent and offspring traits, parent and offspring traits controlling for geographic distances, and parent and offspring traits controlling for environmental distances. Statistics based on Mantel and partial Mantel tests. *r* = standardized Mantel statistic.

Multivariate correlations between	*r*	*P*
Environmental distance vs. geographic distance	0.401	0.083
Parent traits vs. offspring traits	−0.129	0.519
Parent traits vs. offspring traits controlling for geographic distance	−0.203	0.146
Parent traits vs. offspring traits controlling for environmental distance	−0.091	0.412

### Parent and offspring trait–trait correlations

**Figure 6.**
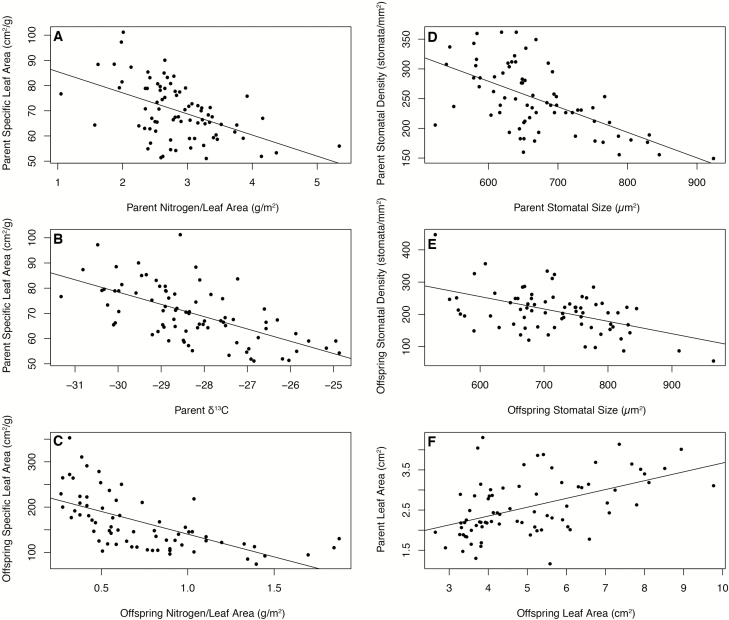
Statistically significant trait–trait linear regressions in parent and offspring populations as well as parent and offspring shared traits.

Trait–trait correlations differed in parent and offspring populations. In parents, apart from the obvious links (e.g. leaf area with SLA, and N_mass_ with C:N and N_area_), SLA was strongly and negatively correlated with N_area_ and δ^13^C ratio (Fig. 6A and B; see **Supporting Information—Tables S6 and S7**). Wood density also correlated with δ^13^C ratio **[see **Supporting Information—Table S7** ]**. In offspring, SLA was strongly and negatively correlated with N_area_ (Fig. 6C; see **Supporting Information—Tables S6 and S7**). Stomata size and stomata density were negatively correlated in both parents and offspring (Fig. 6D and E; see **Supporting Information—Tables S6 and S7**). Of the traits assessed in both parents and offspring **[see **Supporting Information—Table S7** ]**, only leaf area was significantly correlated in parents and offspring (Fig. 6F; see **Supporting Information—Tables S6 and S7**). Mean seed weight did not influence germination, height or relative growth rate **[see **Supporting Information—Table S7** ]**.

## Discussion

Dodonaea displayed significant variation in a broad variety of established functional traits associated with climate over microgeographic distances (from 1.4 to 96.8 km). This variation was likely as a result of acclimation to elevation and/or aridity, and not as a result of genetic differentiation of traits between populations. By studying trait variability across short geographic distances and in a system where environmental and geographic distances were uncorrelated, our results increase our understanding of studying functional trait variability, which is most often done across much larger geographic areas (hundreds of kilometres) (Guerin *et al.* 2012; Hill *et al.* 2015; Baruch *et al.* 2017), and where geography often confounds environmental distance (Meirmans 2015; Caddy-Retalic *et al.* 2017).

We conclude that most observable variation in Dodonaea functional traits was likely a result of environmental acclimation rather than genetic adaptation or differentiation. As such, Dodonaea displays considerable adaptive capacity to acclimate to climate change *in situ*. We therefore suggest that future work on climate adaptation in woody plants should further consider the large amount of trait variation present over short geographic distances, and account for the confounding effect geography has on associating environmental with trait variation. Such robust study designs allow more confident statements about the drivers of adaptive capacity, which is essential to better inform conservation and restoration management strategies, such as designing *in situ* conservation areas and selecting seed for restoration under climate change.

The parent plants in our study system showed great variation in functional traits, with 8 out of 11 differing among populations. Only leaf N content and related did not differ significantly between populations, possibly due to relatively homogeneous site soil N content (Ordoñez *et al.* 2009). A large portion of the phenotypic variability was lost when offspring were grown in the common garden, implying strong plasticity of most traits (only 4 out of 13 of the tested traits differed significantly between offspring populations). Running our common garden experiments in a greenhouse with uniform and favourable growth conditions may have contributed to this high plasticity (Poorter *et al.* 2016). Further experimental work would also assist with teasing plasticity from genetic effects in this system, such as reciprocal transplant studies across the environmental gradient, and comparing trait vs. neutral genetic differentiation (Breed *et al.* 2018). It is also possible that differences in ontogenetic stage between mature individuals and plantlets contributed to their trait differences. Our results were consistent with previous work on *Protea repens*, a sclerophyllous shrub in South Africa, in terms of trait responses to aridity (Carlson *et al.* 2016). We also observed a doubling of SLA and leaf area in the offspring grown in the greenhouse trial, indicating considerable trait plasticity, as previously reported (Zhao *et al.* 2012). Indeed, lower field SLA was probably caused by higher irradiance in conjunction with higher temperatures (Poorter *et al.* 2009,^2014^). However, leaf N_mass_ was significantly lower in offspring; a dilution effect that can potentially be attributed to by rapid growth and expansion of leaves under the favourable growing conditions of the greenhouse.

Despite the climatic and elevational gradients in our study being shorter than those previously sampled (Baruch *et al.* 2017), leaf area and SLA responded in a consistent fashion, where both became smaller under more arid and warmer conditions. This is a common response in plants (Poorter *et al.* 2009; Guerin *et al.* 2012; Guerin and Lowe 2013; Carlson *et al.* 2016), but such a parallel response in leaf area and SLA along short and long environmental clines emphasizes the importance of studying both traits. Small leaves offer better control of temperature, while low SLA provides more efficient use of resources in stressful habitats (Poorter *et al.* 2009). Average seed mass was higher in wetter sites at higher elevation, which was an expected response to more resources and less stressful conditions. Wood usually becomes denser under more stressful environments as narrower xylem vessels control better water flow through the stem (Martínez-Cabrera *et al.* 2009). However, we did not observe a change in wood density across sites, possibly because our regional drought stress cline was relatively weak. Liu and Noshiro (2003) have also previously reported a lack of altitudinal and latitudinal difference in *D. viscosa* wood anatomy.

The leaf δ^13^C ratios indicated less carbon isotope discrimination (i.e. more positive δ^13^C) as climate became more arid, indicating a higher long-term water use efficiency (Farquhar *et al.* 1989). This is the typical response of plants along drought clines (Prentice *et al.* 2011; Schulze *et al.* 2014). Leaf N_mass_ and N_area_ did not respond to the environmental cline, possibly because of an absence of variation in soil N content, as discussed above. However, leaf δ^15^N ratios were positive in the less arid sites, which tend to have higher soil N availability. Nevertheless, δ^15^N is a complex trait that also scales with short-term variation in N cycling and the availability of N supply (Craine *et al.* 2015; Dong *et al.* 2017).

Along our environmental cline, stomatal density decreased but their size increased under cooler and wetter conditions, which is also a common, but environmentally dependant, response in plants (Franks *et al.* 2009; Hill *et al.* 2015; Carlson *et al.* 2016). Small and dense stomata promote transpiration cooling under hotter environments. Adjusting stomatal size and density is a powerful plastic mechanism for thermal regulation and gas exchange in local populations (Franks *et al.* 2009).

Most of the traits surveyed here are part of the ‘leaf economics spectrum’ and, for parent populations, showed the trade-offs consistent with longer and steeper environmental gradients (Wright *et al.* 2004). As such, we were able to compare our results in a well-established empirical and theoretical context. Specific leaf area is prevalent and central among functional traits because it scales positively with growth and photosynthetic rate, and negatively with leaf longevity (Wright *et al.* 2004). Parent SLA was strongly and inversely correlated to the δ^13^C ratio, which is associated with carbon assimilation and long-term water use efficiency (Farquhar *et al.* 1989). Populations with higher SLA displayed higher δ^13^C discrimination, as anticipated for plants under more favourable conditions at higher elevation. Similar results have been observed along aridity gradients (Prentice *et al.* 2011). Also, we found a negative correlation between SLA and N_area_, which is characteristic of fast-growing individuals (Wright *et al.* 2004; Onoda *et al.* 2017). Further, low SLA in dry environments is usually associated with high N_area_ (Dong *et al.* 2017).

The positive association between parent δ^13^C and N_area_ was expected, as high N_area_ enhances photosynthetic capacity reducing internal CO_2_ concentration (*ci:ca*) and increasing δ^13^C (Cernusak *et al.* 2013). The δ^13^C ratio and stomatal density were positively correlated through the direct association of δ^13^C to *ci:ca*, which is regulated by stomatal conductance (Cernusak *et al.* 2013). Therefore, it was expected that the δ^13^C ratio associated with stomatal traits, such as density or size, as well as between stomatal traits and N_area_. Specific leaf area and stomatal density were negatively but weakly correlated, as anticipated from their respective associations with δ^13^C discussed above. Parent populations differed significantly in SLA, which was inversely (although weakly) correlated to wood density, supporting the relationship expected for these traits where faster growing individuals (with high SLA) had low wood density (Chave *et al.* 2009). Also, the δ^15^N ratio and N_area_ were positively correlated, following the trend observed in other studies and attributed to high N availability (Craine *et al.* 2015; Dong *et al.* 2017). In offspring populations, only the correlations between SLA and N_area_, and stomatal density and stomatal size, persisted. The absence of correlations in plants under favourable conditions indicates the importance of resource limitation for maintaining the web of coordinated functional leaf trait responses, and also the high plasticity Dodonaea displays for these traits. At the whole-plant level, the relationship between the reproductive traits (seed mass and germination) and seedling performance (height and relative growth rate) was either absent or reduced, possibly as a result of the environmental uniformity of the common garden.

## Conclusions

Results from our common garden experiment demonstrate that a range of key functional traits in Dodonaea were highly plastic (e.g. SLA, leaf nitrogen content, carbon and nitrogen isotope ratios, stomatal size and density). We sampled populations over microgeographic distances with strong environmental gradients and observed no clear genetic differentiation in most functional traits. As such, Dodonaea has great adaptive potential via functional acclimation, at least in this region. However, in the presence of increased barriers to gene flow with habitat fragmentation (Young *et al.* 1996), as is likely for many Dodonaea populations in this region (Guerin *et al.* 2016), there is a greater chance that the observed plastic trait divergence could accumulate a genetic basis as gene flow is reduced between populations leading to genetic assimilation (Waddington 1953; Pfennig *et al.* 2010). If this process plays out, these populations would likely have a reduced capacity to adapt to climate change due to increased genetic basis in these functional traits and reduced interpopulation gene flow. Future studies in this study system should therefore assess trait plasticity in populations from more fragmented and isolated habitat patches, rather than from the large and intact populations we studied here. In addition, it should be noted that high plasticity, as observed here, may indeed be adaptive in the presence of high environmental variation. Further, since Dodonaea is commonly used for restoration in southern Australia (Monie *et al.* 2013; Pickup *et al.* 2013), and that local populations likely house considerable trait variability via acclimation, the potential risks of transferring seed (Breed *et al.* 2013) across this study region are not likely to be a significant issue.

## Sources of Funding

This work was supported by Australian Research Council funding (DE150100542 awarded to M.F.B.; DP150103414 awarded to M.F.B. and A.J.L.), and a Thomas Davies Research Fund grant from the Australian Academy of Science to M.F.B. (grant number 201408410176).

## Contributions by the Authors

All authors designed the experiments; Z.B., A.R.J., K.E.H., F.A.M., C.B., S.C.-R., M.J.C., N.J.C.G., I.M.-F., K.E.N. and M.F.B. generated and analysed the data; Z.B., A.R.J. and M.F.B. wrote the first draft of the manuscript; and all authors contributed to revisions.

## Conflict of Interest

None declared.

## Supporting Information

The following additional information is available in the online version of this article—

**Appendix S1.** Table of statistical analyses performed throughout the study.

**Figure S1.** Images of *Dodonaea viscosa* (A) seedlings at 3-month-old, (B) at 12-month-old and (C) an adult female bearing ripe fruit in the field.

**Figure S2.** Box plots of offspring traits that displayed significant variation across populations and tested with linear mixed-effects models, with family nested as a random effect within population.

**Table S1.** Linear geographic distances between populations.

**Table S2.** Pearson correlation coefficients of environmental variables and traits of parents and offspring after principal component analysis (PCA) ordination.

**Table S3.** ANOVA table for trait differences between populations for parent and offspring populations.

**Table S4.** Coefficients and significance of the regressions between traits and environmental composite axis PCA1.

**Table S5.** Coefficients and significance of the regressions between traits and environmental composite axes PCA1 and PCA2.

**Table S6.** Trait–trait correlations in parents, offspring and parent–offspring shared traits.

**Table S7.** Pearson correlation coefficients of trait–trait correlations.

Supplementary Information
