# Supplementary material for: Functional acclimation across microgeographic scales in Dodonaea viscosa
Source: AoB Plants. 2018 May 11;10(3):ply029. doi: 10.1093/aobpla/ply029 (PMC6007226; doi:10.1093/aobpla/ply029)
Supplement: Supplementary Information [file ply029_suppl_supplementary_information.docx]

*Supplementary Information*

**Functional acclimation across microgeographic scales in *Dodonaea viscosa***

**Running Title:** Microgeographic variation in functional traits

Appendix S1: Summary of study analyses in relation to research questions.

| **Theme** | **Research question** | **Variable(s)** | **Statistical method** | **Result** |
| --- | --- | --- | --- | --- |
| *Environmental correlation* | Are there clear environmental differences between the 8 sampled populations? | All 7 environmental variables (tested individually across populations) | One-way ANOVA (using Bonferroni correction for multiple testing) | Elevation, aridity index and MAT varied significantly across populations |
|  | Can environmental variation between populations be simplified to new, composite variables? | All environmental variables | Principal Component Analysis (PCA) for environmental space ordination | PC1 (representing climate variables) explained >79% and PC2 explained > 18% of environmental variation across the populations. |
|  | Are parent plant traits correlated with their environment (represented by the composite environmental PCA axes)? | Population environmental PCA1 & PCA2 values, all parent traits | Pearson correlation of PC1/2 with each parent trait | LA & LSA were significantly negatively and δ^15^N & δ^13^C were significantly positively correlated to environmental PC1 (representing AI, MAP & MAT) |
|  | Are offspring plant traits correlated with the environment of their parent population (represented by the composite environmental PCA axes)? | Population environmental PCA1 & PCA2 values, all offspring traits | Pearson correlation of PC1/2 with each offspring trait | Offspring RGR was significantly negatively correlated to environmental PC1 (representing AI, MAP & MAT at the parent population) |
| *Trait correlation* | Is there variation in parent and offspring traits across the 8 sampled populations? | All parent and offspring traits (tested individually) | One-way ANOVA (using Bonferroni correction for multiple testing). Separate linear random-effects models for offspring RGR and HEIGHT (the larger sample size for these traits supported more complex models) | 10 of the 11 tested parent traits were significantly different between populations. Only 4 of the 13 offspring traits (RGR, HEIGHT, LA and germination) varied significantly between populations. |
|  | Do parent and offpring plants from the same population have similar combinations of traits (i.e. do they occupy the same 'trait space'? | All data for traits measured in both parents and offspring (N = 9) | Principal Component Analysis (PCA) for trait space ordination and correlation of Euclidean distance matrices using a partial Mantel test. | Parent plant trait ordination was more structured than offspring trait ordination. There was no correlation between the trait ordinations of parents and offspring from the same population. |
|  | Are parent and offspring traits correlated within each population? | 9 traits measured in both parent and offspring | Pearson correlation among traits for parents and offspring | More significant trait-trait correlations in parents than in offspring. |
|  | Is there correlation between different plant traits and are these common to both parents and offspring? | All parent and offspring traits | Linear regression (with bootstrapping for 95 % confidence intervals and Bonferroni correction for multiple testing). These were run separately for 1) pairs of parent traits, 2) pairs offspring traits and 3) parent & offspring shared traits. | In both parents and offspring, SLA and N_area_, and SD and SS, were significantly negatively correlated. LA was significantly positively correlated between parents and offspring. |

Images of *Dodonaea viscosa* (a) seedlings at 3 month old, (b) at 12 month old, and (c) an adult female bearing ripe fruit in the field. Photo credit: (a) Martin Breed, (b) Zdravko Baruch, (c) Nick Gellie


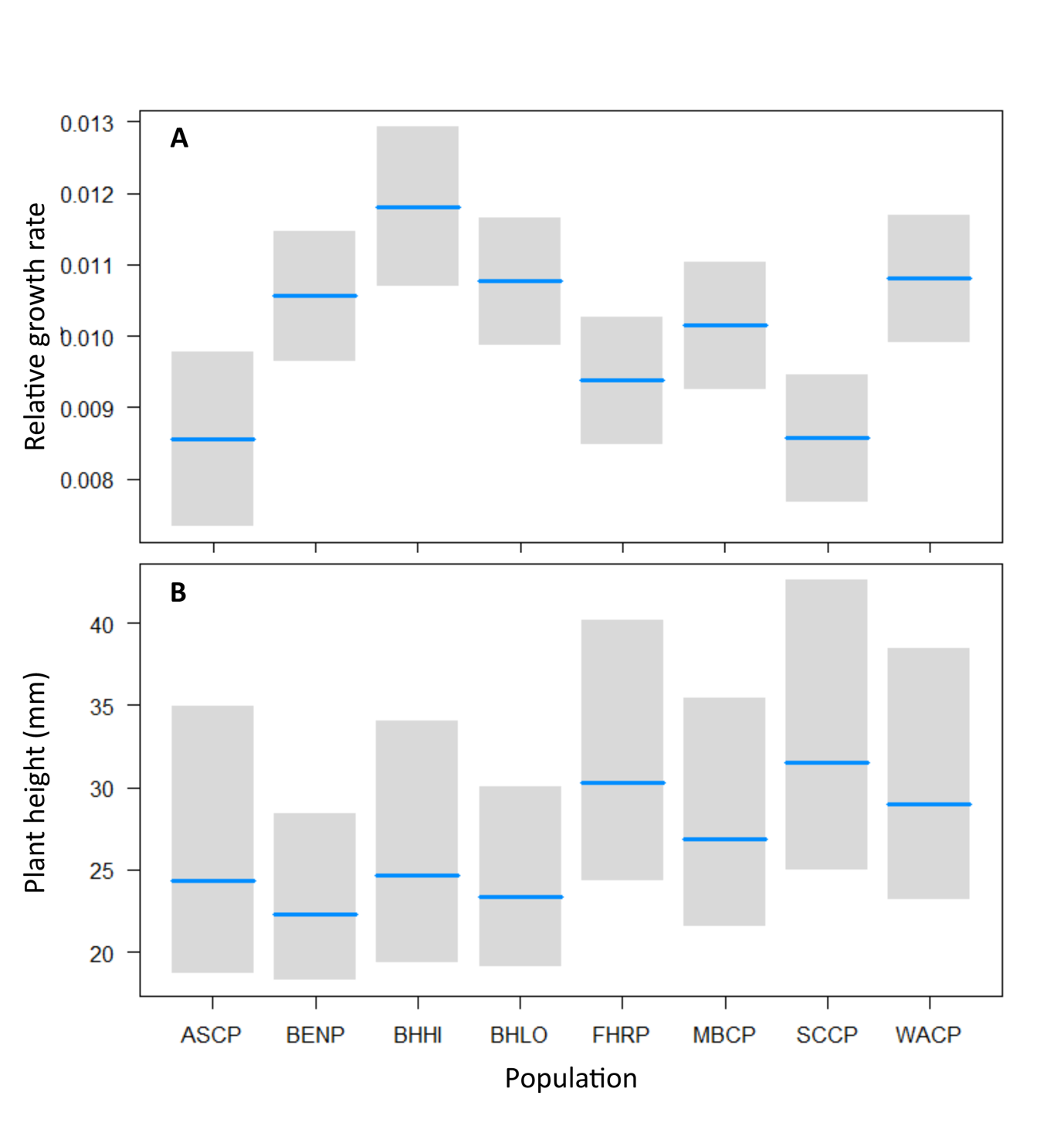


**Figure S1** Box plots of offspring traits that displayed significant variation across populations and tested with linear mixed-effects models, with family nested as a random effect within population.

**Table S1** Linear geographic distances between populations (km).

|  | **ASCP** | **BENP** | **BHHI** | **BHLO** | **FHRP** | **MBCP** | **SCCP** |
| --- | --- | --- | --- | --- | --- | --- | --- |
| **BENP** | 36.55 |  |  |  |  |  |  |
| **BHHI** | 51.03 | 14.47 |  |  |  |  |  |
| **BHLO** | 50.71 | 14.21 | 1.44 |  |  |  |  |
| **FHRP** | 29.84 | 7.14 | 21.35 | 20.93 |  |  |  |
| **MBCP** | 21.66 | 50.1 | 63.76 | 69.88 | 44.9 |  |  |
| **SCCP** | 84.41 | 47.94 | 33.53 | 33.73 | 54.59 | 96.88 |  |
| **WACP** | 75.07 | 38.65 | 24.49 | 25.2 | 45.71 | 85.55 | 13.75 |

**Table S2** Pearson correlation coefficients of environmental variables and traits for both parents and offspring after PCA ordination. Acronyms as in Table 1.

| **Variable** | **PCA1** | **PCA2** |
| --- | --- | --- |
|  | **r** | **r** |
| **Parent environment** |  |  |
| AI | -0.880 | 0.459 |
| MAP | -0.810 | -0.582 |
| MAT | 0.983 | -0.068 |
|  |  |  |
| **Parent** |  |  |
| LA | -0.730 | 0.137 |
| SLA | -0.919 | -0.282 |
| WD | 0.429 | 0.305 |
| SW | 0.209 | -0.863 |
| SD | 0.796 | -0.126 |
| SS | -0.703 | 0.569 |
| δ^13^C | 0.800 | -0.309 |
| δ^15^N | 0.880 | 0.393 |
| N_mass_ | -0.539 | -0.648 |
| N_area_ | 0.788 | -0.072 |
|  |  |  |
| **Offspring** |  |  |
| GERM | -0.465 | -0.095 |
| HEIGHT | -0.458 | 0.296 |
| RGR | -0.006 | -0.927 |
| SD | 0.706 | -0.152 |
| SS | -0.488 | -0.574 |
| LA | -0.920 | 0.143 |
| SLA | -0.643 | -0.620 |
| THICK | -0.731 | -0.006 |
| δ^13^C | -0.474 | 0.015 |
| δ^15^N | 0.374 | 0.112 |
| N_mass_ | -0.438 | 0.886 |
| C:N | 0.077 | -0.676 |
| N_area_ | 0.100 | 0.473 |

**Table S3**. ANOVA table for trait differences between parent and offspring populations. Results for relative growth rate (RGR) and plant height (HEIGHT) of offspring are within text and both are statistically significant. Acronyms as in Table 1.

| **Traits** | **Parents** | | |  | **Offspring** | | |
| --- | --- | --- | --- | --- | --- | --- | --- |
|  | df | F | P |  | df | F | P |
| **LA** | (7,72) | 6.27 | <0.0001 |  | (7,72) | 5.68 | <0.0001 |
| **SLA** | (7,72) | 7.95 | <0.0001 |  | (7,68) | 1.59 | 0.151 |
| **SD** | (7,67) | 5.54 | <0.0001 |  | (7,64) | 1.65 | 0.135 |
| **SS** | (7,67) | 6.55 | <0.0001 |  | (7,64) | 1.63 | 0.141 |
| **δ^13^C** | (7,72) | 9.04 | <0.0001 |  | (7,66) | 1.94 | 0.077 |
| **δ^15^N** | (7,72) | 32.4 | <0.0001 |  | (7,66) | 0.74 | 0.630 |
| **N_mass_** | (7,72) | 0.85 | 0.550 |  | (7,66) | 0.54 | 0.802 |
| **C:N** | (7,72) | 1.06 | 0.398 |  | (7,66) | 0.97 | 0.459 |
| **N_area_** | (7,72) | 1.35 | 0.236 |  | (7,66) | 1.54 | 0.171 |
| **WD** | (7,72) | 17.3 | <0.0001 |  | - | - | - |
| **SW** | (7,72) | 2.62 | <0.05 |  | - | - | - |
| **GERM** | - | - | - |  | (7,72) | 7.04 | <0.0001 |
| **THICK** | - | - | - |  | (7,55) | 0.91 | 0.500 |

**Table S4.** Significant regression coefficients of parent and offspring traits with PCA1 with threshold P value adjusted by sequential Bonferroni. For the significant correlations, the linear model was run with 10000 bootstrap iterations to derive 95%CI around the slope. Acronyms as in Table 1.

| **Trait** |  |  | **r** | **P observed** | **Threshold P** | **n** | **ß (bootstrap 95%CI)** |
| --- | --- | --- | --- | --- | --- | --- | --- |
| **Parents** |  |  |  |  |  |  |  |
| SLA |  |  | -0.387 | 0.0001 | 0.00096 | 80 | -2.962 (-4.372, -1.456) |
| LA |  |  | -0.366 | 0.001 | 0.00098 | 80 | -0.181 (-0.280, -0.071) |
| δ^15^N |  |  | 0.327 | 0.003 | 0.001 | 80 | 0.396 (0.144, 0.601) |
|  |  |  |  |  |  |  |  |
| **Offspring** |  |  |  |  |  |  |  |
| RGR |  |  | -0.327 | 0.003 | 0.00384 | 80 | -0.0005 (-0.0008, -0.0002) |

**Table S5** Coefficients and significance of the regressions between all traits and PCA1 and PCA2 of parent and offspring populations along the composite environmental axis. Significant correlations (P<0.05) are in bold. Acronyms as in Table 1.

|  | **PCA1** | | **PCA2** | |
| --- | --- | --- | --- | --- |
|  | **r** | **P** | **r** | **P** |
| **PARENTS** |  |  |  |  |
| LA | **-0.366** | **<0.001** | 0.164 | 0.153 |
| SLA | **-0.387** | **<0.0001** | **-0.396** | **<0.0001** |
| WD | 0.158 | 0.159 | 0.243 | **<0.05** |
| SW | **-0.224** | **<0.05** | 0.110 | 0.328 |
| SD | 0.032 | 0.890 | 0.148 | 0.210 |
| SS | 0.114 | 0.336 | **0.338** | **<0.01** |
| δ^13^C | **0.302** | **<0.01** | **0.410** | **<0.0001** |
| δ^15^N | **0.327** | **<0.01** | 0.095 | 0.402 |
| N_mass_ | 0.158 | 0.162 | 0.032 | 0.974 |
| C:N | 0.176 | 0.117 | 0.045 | 0.686 |
| N_area_ | 0.118 | 0.288 | **0.261** | **<0.05** |
|  |  |  |  |  |
| **OFFSPRING** |  |  |  |  |
| LA | 0.032 | 0.856 | **-0.279** | **<0.05** |
| SLA | **0.237** | **<0.05** | 0.045 | 0.708 |
| SD | 0.173 | 0.147 | 0.032 | 0.769 |
| SS | 0.138 | 0.245 | 0.010 | 0.905 |
| δ^13^C | 0.032 | 0.897 | 0.212 | 0.069 |
| δ^15^N | 0.071 | 0.570 | 0.187 | 0.110 |
| N_mass_ | 0.173 | 0.141 | 0.063 | 0.594 |
| C:N | 0.161 | 0.169 | 0.045 | 0.696 |
| N_area_ | 0.134 | 0.240 | 0.045 | 0.668 |
| GERM | 0.063 | 0.570 | 0.179 | 0.114 |
| HEIGHT | 0.187 | 0.100 | 0.210 | 0.067 |
| RGR | **-0.327** | **<0.01** | 0.221 | 0.051 |
| THICK | 0.063 | 0.621 | 0.010 | 0.921 |

**Table S6**. Trait-trait correlations in parents, offspring and parent-offspring shared traits with threshold P value adjusted by sequential Bonferroni. For the significant correlations, the linear model was run with 10000 bootstrap iterations to derive 95%CI around the slope. Parent wood density and δ^15^N were omitted as they were not considered to be ecologically relevant. Acronyms as in Table 1.

| **Trait 1** | **Trait 2** | **r** | **P observed** | **Threshold P** | **n** | **ß (bootstrap 95 CI)** |
| --- | --- | --- | --- | --- | --- | --- |
| **Parents** |  |  |  |  |  |  |
| SLA | N_area_ | -0.49 | <0.00001 | 0.000961 | 80 | -8.378 (-12.207, -5.384) |
| SLA | δ^13^C | -0.62 | <0.00001 | 0.000980 | 80 | -4.878 (-6.108, -3.768) |
| SD | SS | -0.59 | <0.00001 | 0.001 | 72 | -0.430 (-0.547, -0.313) |
|  |  |  |  |  |  |  |
| **Offspring** |  |  |  |  |  |  |
| SD | SS | -0.49 | <0.00001 | 0.000581 | 72 | -0.384 (-0.568, -0.1871) |
| SLA | N_area_ | -0.45 | <0.00001 | 0.000581 | 70 | -100.23 (-138.77 -72.10) |
|  |  |  |  |  |  |  |
| **Parents** | **Offspring** |  |  |  |  |  |
| LA | LA | 0.49 | <0.00001 | 0.000324 | 77 | 0.220 (0.145, 0.296) |

**TABLE S7.** Pearson correlation coefficients of trait-trait correlations within parents, offspring, and parents-offspring. Coefficients in bold indicate P < 0.05. Acronyms as in Table 1.

| **Parent-Parent** | **SLA** | **WD** | **SW** | **SD** | **SS** | **δ^13^C** | **δ^15^N** | **N_mass_** | **C:N** | **N_area_** |  |  |
| --- | --- | --- | --- | --- | --- | --- | --- | --- | --- | --- | --- | --- |
| **LA** | **0.339** | 0.141 | 0.245 | **-0.272** | **0.292** | 0.2 | **-0.395** | 0.1 | 0.032 | 0.145 |  |  |
| **SLA** |  | 0.176 | 0.032 | **-0.286** | 0.134 | **-0.618** | **-0.346** | 0.19 | **-0.237** | **-0.492** |  |  |
| **WD** |  |  | 0.032 | 0.095 | 0.077 | **0.375** | **0.336** | **-0.265** | 0.214 | 0.118 |  |  |
| **SW** |  |  |  | 0.105 | 0.063 | 0.045 | 0.158 | 0.187 | 0.207 | 0.152 |  |  |
| **SD** |  |  |  |  | **-0.593** | **0.303** | **0.354** | 0.145 | 0.089 | 0.077 |  |  |
| **SS** |  |  |  |  |  | 0.182 | **-0.307** | 0.032 | 0.055 | 0.118 |  |  |
| **δ^13^C** |  |  |  |  |  |  | **0.389** | 0.063 | 0.1 | **0.341** |  |  |
| **δ^15^N** |  |  |  |  |  |  |  | 0.11 | 0.145 | **0.311** |  |  |
| **N_mass_** |  |  |  |  |  |  |  |  | **-0.944** | **0.746** |  |  |
| **C:N** |  |  |  |  |  |  |  |  |  | **-0.662** |  |  |
|  |  |  |  |  |  |  |  |  |  |  |  |  |
| **Offspring-Offspring** | **SLA** | **THICK** | **GERM** | **HEIGHT** | **RGR** | **SD** | **SS** | **δ^13^C** | **δ^15^N** | **N_mass_** | **C:N** | **N_area_** |
| **LA** | **0.511** | 0.071 | 0.212 | 0.155 | 0.063 | 0.114 | **0.373** | 0.032 | 0.13 | 0.045 | 0.045 | **-0.247** |
| **SLA** |  | 0.032 | 0.187 | 0.118 | 0.2 | 0.055 | 0.122 | 0.122 | 0.122 | 0.032 | 0.045 | **-0.453** |
| **THICK** |  |  | 0.134 | 0.105 | 0.11 | 0.095 | 0.228 | 0.122 | 0.138 | **-0.356** | **0.311** | 0.197 |
| **GERM** |  |  |  | **0.286** | 0.122 | 0.032 | 0.032 | 0.032 | 0.032 | 0.084 | 0.077 | 0.071 |
| **HEIGHT** |  |  |  |  | 0.032 | 0.095 | 0.195 | 0.126 | 0.063 | 0.032 | 0.063 | 0.045 |
| **RGR** |  |  |  |  |  | 0.032 | 0.055 | 0.077 | 0.032 | 0.11 | 0.071 | 0.126 |
| **SD** |  |  |  |  |  |  | **-0.494** | **0.335** | 0.152 | 0.095 | 0.055 | 0.032 |
| **SS** |  |  |  |  |  |  |  | **-0.339** | **-0.302** | **-0.338** | **0.401** | **-0.344** |
| **δ^13^C** |  |  |  |  |  |  |  |  | **0.423** | **0.239** | **-0.303** | 0.126 |
| **δ^15^N** |  |  |  |  |  |  |  |  |  | 0.221 | **-0.276** | 0.126 |
| **N_mass_** |  |  |  |  |  |  |  |  |  |  | **-0.909** | **0.659** |
| **C:N** |  |  |  |  |  |  |  |  |  |  |  | **-0.565** |
|  |  |  |  |  |  |  |  |  |  |  |  |  |
| **Parent-Offspring** | **LA** | **SLA** | **GERM** | **HEIGHT** | **RGR** | **SD** | **SS** | **δ^13^C** | **δ^15^N** | **N_mass_** | **C:N** | **N_area_** |
| **LA** | **0.493** | **0.336** | **0.268** | 0.032 | 0.11 | 0.089 | **0.243** | 0.032 | 0.032 | 0.1 | 0.17 | **-0.27** |
| **SLA** | 0.152 | 0.032 | 0.032 | 0.032 | 0.032 | 0.105 | 0.134 | 0.158 | 0.148 | 0.032 | 0.077 | 0.032 |
| **WD** | **-0.274** | **-0.268** | 0.095 | **0.285** | **-0.253** | 0.1 | 0.032 | 0.032 | 0.145 | 0.045 | 0.045 | 0.21 |
| **SW** | 0.032 | 0.141 | 0.055 | 0.1 | 0.192 | **0.243** | 0.032 | 0.17 | 0.167 | 0.1 | 0.11 | 0.032 |
| **SD** | **-0.272** | 0.045 | 0.217 | 0.045 | 0.045 | 0.224 | 0.148 | 0.032 | 0.21 | 0.095 | 0.032 | 0.077 |
| **SS** | **0.373** | 0.1 | 0.032 | **0.355** | 0.217 | 0.173 | **0.245** | 0.11 | 0.155 | 0.045 | 0.032 | 0.11 |
| **δ^13^C** | **-0.295** | **-0.263** | 0.055 | 0.084 | 0.045 | 0.084 | 0.055 | 0.118 | 0.214 | 0.105 | 0.1 | 0.077 |
| **δ^15^N** | **-0.459** | 0.173 | 0.118 | 0.138 | 0.032 | 0.032 | 0.032 | 0.055 | 0.192 | 0.032 | 0.032 | 0.114 |
| **N_mass_** | 0.063 | 0.077 | 0.164 | 0.134 | 0.145 | 0.089 | 0.055 | 0.032 | 0.032 | 0.077 | 0.032 | 0.084 |
| **C:N** | 0.071 | 0.1 | 0.167 | 0.084 | 0.089 | 0.089 | 0.032 | 0.032 | 0.032 | 0.071 | 0.032 | 0.063 |
| **N_area_** | 0.045 | 0.063 | 0.158 | 0.063 | 0.11 | 0.032 | 0.032 | 0.182 | 0.077 | 0.045 | 0.055 | 0.032 |
